# Factors Associated with the Remission of Type 1 Diastolic Dysfunction after Dapagliflozin Treatment in Patients with Type 2 Diabetes

**DOI:** 10.3390/jcm9113779

**Published:** 2020-11-23

**Authors:** Adina Braha, Alin Albai, Romulus Timar, Laura Diaconu, Lucian Vasiluță, Daniela Cipu, Bogdan Timar, Alexandra Sima

**Affiliations:** 1 Second Department of Internal Medicine, “Victor Babeș” University of Medicine and Pharmacy, 300041 Timisoara, Romania; braha.adina@umft.ro (A.B.); timar.romulus@umft.ro (R.T.); diaconu.laura@umft.ro (L.D.); l.vasiluta@yahoo.com (L.V.); bogdan.timar@umft.ro (B.T.); alexa_moisuc@yahoo.com (A.S.); 2 Department of Orthopedics-Traumatology, Urology and Medical Imaging, “Victor Babeș” University of Medicine and Pharmacy, 300041 Timisoara, Romania; danscipu@yahoo.com

**Keywords:** type 1 diastolic dysfunction, dapagliflozin, heart failure, liver steatosis, liver fibrosis

## Abstract

Patients with type 2 diabetes (T2DM) are at high risk of developing cardiovascular disease and heart failure (HF), both with preserved and reduced ejection fraction of the left ventricle. Previous research demonstrated that dapagliflozin treatment is associated with the remission of type 1 diastolic dysfunction (DD1) in patients with T2DM. The main aim of this study was to evaluate the possible baseline predictors associated with the remission of DD1 in patients with T2D after one year of dapagliflozin treatment. In this prospective and observational study, 45 patients with T2DM were evaluated before and after one year of treatment with 10 mg dapagliflozin daily added to their background therapy. In the studied group, 73.3% (33/45) of the patients had DD1 at baseline. The primary outcome of this research was DD1 remission. DD1 remission was associated with improvement of liver stiffness, an increase in estimated glomerular filtration rate (eGFR), and a decrease in hemoglobin A1c (HbA1c). Independent predictors for the remission of DD1 were a more than 0.4 kPa difference in the initial stiffness score and the 1-year assessment fibrosis score and a duration of diabetes ≤8 years. Age, body mass index (BMI), or patient weight after one year did not influence the DD1 outcome. Patients with a T2DM duration of less than eight years have the additional benefit of DD1 remission associated with dapagliflozin treatment beyond the conventional benefits such as improvements in glycemic control, cardiovascular, renal, and hepatic risk reductions. In patients with T2DM, the remission of DD1 was associated with decrease of liver stiffness.

## 1. Introduction

Diabetes mellitus (DM) is a significant risk factor for developing cardiovascular disease, which is the leading cause of death among patients with diabetes [1], and an independent cause for heart failure (HF) even in the absence of coronary atherosclerosis, hypertension, or other conditions traditionally associated with HF [2,3]. HF is a syndrome characterized by non-specific and various clinical signs, which makes the management of this disease a challenge in terms of early diagnosis and treatment. 

The diastolic dysfunction of the left ventricle is a pathological condition preceding left HF, often asymptomatic and insufficiently diagnosed, characterized by altered relaxation and compliance of the left ventricular myocardium [4]. In type 1 diastolic dysfunction (DD1), impaired relaxation of the left ventricle is found; in contrast to other grades of diastolic dysfunction, DD1 is reversible [5]. Previous research demonstrated that left ventricular diastolic dysfunction is a strong predictor for cardiovascular outcome, even in patients with reduced ejection fraction [6,7].

From a historical perspective, for several decades, the pharmacotherapy of T2DM was focused on reducing blood glucose, based on the idea that intensive glycemic control will bring micro- and macrovascular benefits. Large clinical trials demonstrated a link between glycemic control (evaluated by glycated hemoglobin—HbA1c) and the incidence of chronic complications of diabetes, highlighting the beneficial effect of strict glycemic control on microvascular complications [8]. However, strict glycemic control was not consistently associated with a decrease in the incidence of major cardiovascular events or with an improvement regarding survival after myocardial infarction or stroke [9,10,11]. 

Therefore, the interest in novel antidiabetic therapies, such as sodium-glucose co-transporter-2 inhibitors (SGLT2i), has been growing. This class of drugs increases urinary glucose excretion independently of insulin secretion and causes weight loss, in addition to numerous other pleiotropic effects, resulted beyond anti-hyperglycemic efficacy [12]. Dapagliflozin, a SGLT2i, has been noted for its ability to reduce heart failure aggravation or cardiovascular-related death, regardless of the stage of diabetes [13].

The main aim of this study was to evaluate the possible baseline predictors associated with the remission of DD1 in patients with T2DM after one year of treatment with dapagliflozin.

## 2. Material and Methods

### 2.1. Study Design and Patients

In this prospective, observational study, 80 patients with T2DM attending scheduled medical visits at the Diabetes Clinic and Diabetes Outpatient of “Pius Brînzeu” Emergency Hospital were enrolled. Physicians prescribing diabetes treatment referred T2DM patients for this study before the initiation of dapagliflozin treatment. The ethical committee of the “Pius Brînzeu” Emergency County Hospital approved this research and it has met the requirements of the Helsinki Declaration. All participants agreed and signed a written informed consent before enrollment in the study. 

T2DM patients over 18 years old, without cardiac valvulopathies or significant cardiac disease, without chronic viral hepatitis, with no alcohol misuse, with HbA1c > 7%, left ventricular ejection fraction (LVEF) > 40%, and an estimated glomerular filtration rate (eGFR) > 60 mL/min/1.73m^2^, met the eligibility criteria for participating in the study. Exclusion was based on the following criteria: LVEF < 40% and significant structural heart disease, ongoing or planned pregnancy, lactation, other than type 2 diabetes types, history of diabetic ketoacidosis, HbA1c < 7%, eGFR < 60 mL/min/1.73m^2^, normal-weight with a BMI < 25kg/m^2^, urinary tract infections that occurred during the study (possibly secondary to dapagliflozin treatment), or the patients refuse to participate in this clinical trial. 

### 2.2. Methods

During the study, some patients were excluded due to intolerance or inefficacy of the treatment or at their request. We performed complex investigations in 45 patients enrolled in the study before starting daily treatment with 10 mg of dapagliflozin. Dapagliflozin was add-on therapy to metformin, sulfonylureas or insulin as follows: 66.7% of the included patients had treatment with metformin, 11.1% had therapy with metformin and sulfonylureas, 11.1% had basal insulin and metformin, 8.9% had sulfonylureas, and 2.2% had intensive insulin therapy without metformin. None of the patients were treated with glucagon-like peptide-1 (GLP1) agonists in the study. We scheduled the follow-up medical examinations at 3, 6 and 12 months, respectively. 20% (9/45) of patients needed an intensification of antidiabetic therapy as recommended by the international guidelines [14] with a molecule containing a combination of dapagliflozin and dipeptidyl-peptidase-4 inhibitors (DPP4i). At the six-month follow-up, patients successfully repeated the initial investigations, and at the one-year follow-up, they repeated only the cardiac ultrasound to screen for type 1 diastolic dysfunction (DD1) and body weight assessment. 

The assessments of clinical status, medical history, biological markers, and paraclinical investigations (cardiac ultrasound, transient elastography with controlled attenuation parameter (CAP), native computed tomography) are described in a previously published paper [15]. Type 1 diastolic dysfunction was defined as E/A < 1 and E/e’ > 15 [5]. We considered as study outcome DD1 remission at one-year medical examination. 73.3% of patients were initially diagnosed with DD1, and after a one-year treatment, only 24.4% remained with DD1 [15].

### 2.3. Statistical Analysis

We performed statistical analysis with GNU PSPP (Version 1.4.1, Software Foundation, Boston, MA, USA), MedCalc Statistical Software version 14.8.1 (MedCalc Software bvba, Ostend, Belgium), and Microsoft Office Excel (Professional Plus 2019 Edition). The distribution of numerical variables was tested with the Kolmogorov–Smirnov test. Continuous numerical variables are presented as mean ± standard deviation, and numerical variables with nonparametric distribution are presented as median and interquartile range. The qualitative/nominal variables are presented as absolute frequencies in the class and relative frequencies (percentages of the total subgroup). To evaluate the significances of the differences, t-test (for normal distribution) and Mann–Whitney U-test (for non-normal distribution) were used. A *p*-value < 0.05 was considered the threshold for statistical significance, and 95% populational confidence intervals were calculated.

To evaluate the impact of numerical and continuous variables on dichotomous events, we built univariate and multivariate logistic regression models. The variation of the dichotomous event’s risk of occurrence was interpreted by the exponent means of B coefficient of the regression equation. 

Linear regression analyses were performed to assess the impact of the variation of biological and paraclinical variables on the study outcomes after dapagliflozin therapy. The dependent variable was DD1 remission, and the independent variables, coded with 1 for positive, 0 for negative, were the following: HbA1c target < 7%; increased high-density lipoprotein cholesterol (HDLc); increased eGFR; decreased values of the following parameters: total cholesterol (CT), low-density lipoprotein cholesterol (LDLc), triglycerides (TG), urinary albumin to creatinine ratio (UACr), uric acid, L4 visceral abdominal fat volume, epi-cardiac fat volume, mediastinal fat thickness, and HbA1c, respectively; improvement of liver fibrosis; improvement of hepatic steatosis at six-month follow-up; bodyweight at six-month follow-up. To evaluate the association of DD1 remission with the factors included in the analysis, measured on a continuous scale (such as patient’s age, diabetes duration, Δ variables between baseline and 6-month follow-up for clinical, biological, and imaging parameters such as BMI, HbA1c, fat volumes, CAP, liver stiffness), we built univariate, then multivariate logistic regression models, with potential predictors—the variables mentioned above, respectively, and DD1 remission as the outcome, after one year of treatment. 

To evaluate the strength of the relationship between a potential etiological factor (the predominant type of fat tissue, gender) and the occurrence of the study outcome, we calculated the relative risk, odds ratio, and correlation coefficient, and the statistical significance of the association between the exposure factor, and the effect is described by the value of p and the 95% confidence interval.

For evaluating the predictive power of predicting DD1 remission based on the Δ stiffness and the age of diabetes, we performed Receiver-Operating Characteristics (ROC) analyses. Predictive performance is described through sensitivity, specificity, and predictive values (positive and negative). The optimal threshold value of the predictor was considered equal to the Youden index. We compared the area under the model’s ROC curve with the non-discriminant one to analyze the predictive capacity’s statistical significance (the area under the ROC curve = 0.5).

## 3. Results

In the study group, 53.3% (24/45) were men. The mean age of the patients was 56.9 ± 9.7 years, and the diabetes duration ranged from 0 to 24 years, with a median of 7 years. The mean age had no significant differences in relation to gender (57.8 ± 8.5 years in men and 55.9 ± 11.1 years in women). Men had a longer diabetes duration than women (10 ± 5.1 years vs. 6.6 ± 4.8 years, *p* = 0.02). The quality of metabolic control, as evaluated by HbA1c, was similarly improved after six months of treatment both in women (ΔHbA1c = 1.2 ± 2.7 percentage points) and in men (ΔHbA1c = 1.3 ± 2.6 percentage points), *p* = 0.9. The dynamics of clinical, biological, and imagistic parameters of the subjects included in this study are presented in Table 1.

The patients who needed an intensification of antidiabetic therapy at 3-month follow-up with a molecule containing a combination of dapagliflozin and DPP4 inhibitors, which constituted 20% of the total subjects (9/45, 4 women, and 5 men), had a mean age of 61.6 ± 9.1 years and a diabetes duration of 9 ± 4.5 years. The main characteristics in dynamics of this subgroup were similar to the initial group.

### 3.1. Binary Regression Models

In the univariate binary regression analysis, DD1 remission is associated with the improvement of liver fibrosis, the increase of eGFR, and the decrease of HbA1c. 

In the multivariate binary regression model, the increase of eGFR and the decrease of HbA1c were kept as significant associations with our outcome (*p* = 0.003, 77.78% correctly classified cases; for each, B coefficient was 2.07, and the standard error 0.96).

The ΔHbA1c between baseline and six-month measurements was significantly associated with the increase of the probability of DD1 remission (*p* = 0.03). The ΔBMI between baseline and the one-year-evaluation reduces the chance of DD1 remission but not statistically significant (*p* = 0.06). Similarly, ΔGFR in the two evaluations indicated that for each decrease of eGFR by 1 mL/min/1.73 m^2^ the probability of DD1 remission decreases by 9% (*p* = 0.02). The patients’ age was not statistically significantly associated with DD1 dynamics (*p* = 0.3). On the same note, patients who had a long diabetes duration were at higher risk of diastolic dysfunction persistence for each year of disease duration (*p* = 0.01), so every year of diabetes will decrease the probability of DD1 remission by 19%. The exponent means of B coefficient of the regression equations are listed in Table 2.

### 3.2. Logistic Regression Models

According to the univariate logistic regression model (Table 2), we observed that the Δ liver stiffness score after six months of dapagliflozin is a statistically significant predictive factor (*p* = 0.04). The ΔCAP was not significantly associated with DD1 remission (*p* = 0.4).

**Figure 1:**
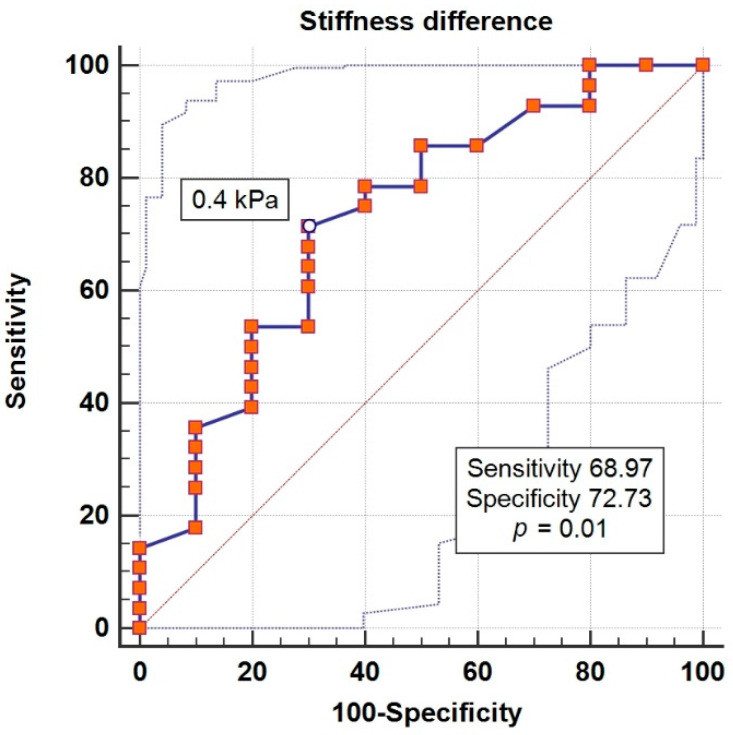
Graphical representation of the Receiver-Operating Characteristics (ROC) curve of the difference in liver stiffness for the prediction of diastolic dysfunction remission.

ROC curve analysis for Δ liver stiffness showed an area under the ROC curve = 0.726, sensitivity 71.4%, specificity 70%, positive predictive values 81.5 and negative 45.5, *p* = 0.02 with Youden Index J = 0.41 (Figure 1). 

**Figure 2:**
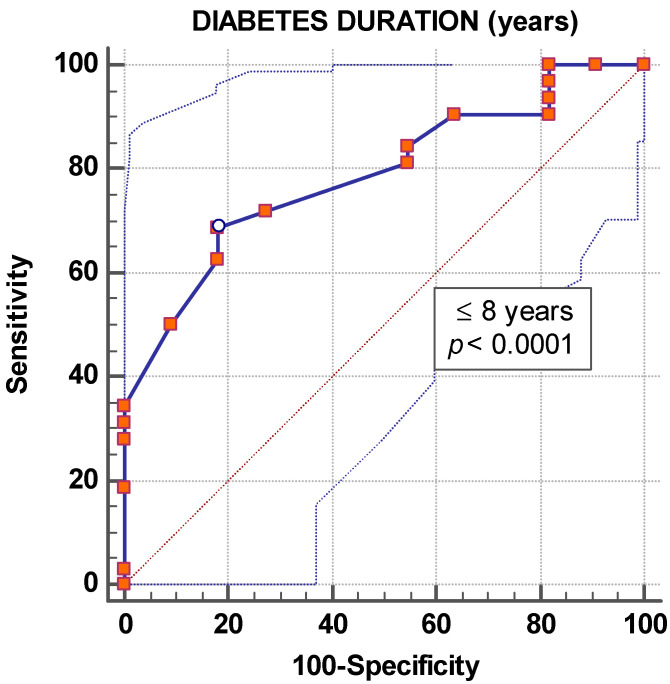
Graphical representation of the ROC curve of diabetes duration for the prediction of diastolic dysfunction remission.

A diabetes duration ≤ 8 years represents a statistically significant predictive factor for DD1 remission, with a sensitivity of 68.7% and specificity of 81.8%, according to the ROC curve (area under the curve 0.788, 91.6 positive predictive values, and 47.3 negative predictive values, *p* < 0.0001), as illustrated in Figure 2.

### 3.3. Risk Analysis

**Figure 3:**
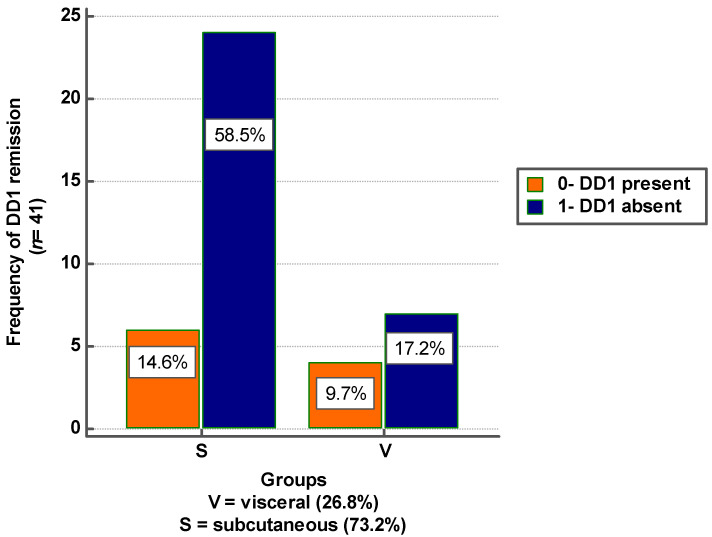
Frequency of diastolic dysfunction remission after one year of dapagliflozin, depending on the predominant type of adipose tissue.

To analyze whether the predominant type of adipose tissue is a risk factor for the DD1 remission following one-year treatment with dapagliflozin, we used the following codes: patients with a subcutaneous/visceral fat ratio greater than 1 were coded with 0, and the others with 1; the patients with remitted DD1 were coded with 1, and the others with 0. Figure 3 illustrates the frequency of DD1 remission stratified by the predominant type of adipose tissue.

We considered the exposure factor the predominant type of adipose tissue, and the expected result DD1 remission. The results were not statistically significant and are presented in Table 3.

To see if the patient’s gender influenced the study outcome, we performed the risk analysis in dynamic, considering the female gender as the risk factor. At the first cardiac ultrasound, women had a relative risk < 1 (RR = 0.42, 95% CI 0.13–1.40), without statistical significance. At one-year follow-up, the analysis indicated a relative risk of > 1 (RR = 1.26, 95% CI 0.89–1.78), but it was not statistically significant.

## 4. Discussion

According to the SHORTWAVE study, in a T2DM population with no documented cardiovascular disease and no signs of ischemia in stress test, left ventricle dysfunction has a high prevalence, up to 68% [16]. In previous studies, diastolic dysfunction has been associated with aging, a long duration of diabetes, increased blood pressure, and interventricular septal thickness, dyslipidemia, and a moderately high HbA1c [17]. Also, there is evidence that the epi-cardiac adipose tissue associated with insulin resistance and coronary artery disease alters the diastolic function by secreting some pro-inflammatory cytokines [18].

In T2DM patients, the prevalence of diastolic dysfunction varies from 47% to 71% because of different echocardiographic criteria used for screening, patients’ cardiac function, and different diastolic dysfunction grades [19,20,21,22]. Similar to other studies, in our research 73% of patients had diastolic dysfunction. However, our screening was specifically addressed for DD1, which we consider of scientific interest because it represents an alteration in the left ventricle’s active relaxation without myocardial structural damage. The presence of myocardial structural changes, atrial fibrillation, or significant valvulopathies were exclusion criteria in this study. Our experiment showed an improvement in the diastolic function after six months of dapagliflozin therapy, but especially after 48 weeks of treatment. From an initial percentage of 73%, at the final cardiac ultrasound evaluation, DD1 was present only in 24.4% of patients. During the study, patients who required intensification of diabetes treatment through the association of a SGLT2i and a DPP4i had the most significant benefit regarding DD1 remission. In these patients, DD1 remission was statistically significant at a 100% rate, while in the group of patients treated only with SGLT2i, the remission rate was 58% [15]. This diastolic improvement may be attributed to the improvement of cardiac output, reduced plasma volume, followed by SGLT2 inhibition [23,24].

In a study by Matsutani et al., screening for diastolic dysfunction was performed on 34 patients with T2DM before and after three months of therapy with canagliflozin; changes in the septal E/e’ ratio was observed. Of all potential predictive factors analyzed with the target of decreasing E/e’, only changes in hemoglobin was associated independently in a multivariate regression model. It is important to note that about 32% of patients evaluated by Matsutani et al. had cardiovascular disease [25].

In our study, the epicardial fat volume and the left atrium volume were associated with the mitral E/A ratio correction. Verma et al. reported similar effects of empagliflozin regarding cardiac structure and heart function in a group of 10 patients with known cardiovascular disease for a short period of 3-month follow-up. They noted a significant reduction in the left ventricle mass index and an improvement in diastolic function, evaluated by e’ wave [26].

In the regression models, we analyzed the extent to which the dynamics of the values of all the reviewed markers influenced our primary end-point—DD1 remission. In univariate binary logistic regression models, the improvement of liver stiffness, the increase in eGFR, and the decrease of HbA1c after six months of therapy with dapagliflozin were associated independently with the DD1 remission. The magnitude of HbA1c decrease after SGLT2i treatment could explain the DD1 remission. Also, it appears that the deterioration of kidney function in patients treated with dapagliflozin could be associated with DD1 persistence. For each decrease of eGFR by 1 mL/min, the risk of DD1 persistence will increase by 9%. In logistic regression analysis, the difference in stiffness score was a predictive factor for DD1 remission, and a reduction of 0.4 kPa in stiffness score could increase the probability of DD1 remission by 24%. After assessing the relationship between potential predictive factors and their simultaneous impact on the improvement of diastolic function, in the multivariate analysis only the stiffness difference after SGLT2i remained a valid, statistically significant predictive factor.

Although the mechanisms underlying these associations are unclear, the present study findings suggest the anti-inflammatory effect of dapagliflozin. In experimental studies, dapagliflozin has been shown to slow glomerulosclerosis and liver fibrosis progression by reducing tissue inflammation markers and oxidative stress induced by chronic hyperglycemia [27]. 

Subjects’ age or gender did not influence the dynamics of DD1. On the contrary, every year of diabetes duration decreased the chance of DD1 remission by 19%. A threshold value of ≤8 years of diabetes duration was a predictive factor for DD1 remission in the ROC analysis. This finding suggests the importance of initiating treatment with dapagliflozin as early as possible.

Studies have concluded that obesity is another significant cause of HF with preserved ejection fraction, hypertension, and DM [28]. Ichikawa et al. demonstrated that abdominal visceral adiposity, measured by computed tomography, was an independent determinant of the parameters of left ventricular diastolic function (E/A, e’, and E/e’) in 148 asymptomatic DM patients with preserved LVEF [29]. Our study results show that DD1 remission was not due to patients’ weight loss. Neither body weight nor BMI was associated with our objective. It is known that obesity may be due to an excess of subcutaneous or visceral adipose tissue. In our study, most patients had subcutaneous excess fat tissue. The predominant type of adipose tissue in these patients did not affect our outcome.

Previous studies have reported an association between diastolic dysfunction and liver stiffness [30] that may be explained by the altered hemodynamics secondary to progressive cardiac fibrosis and diastolic dysfunction which in turn promotes the progression in liver stiffness and fibrosis [31]; liver fibrosis activates cardiac fibrosis as described in a study about ‘cirrhotic cardiomyopathy’, a pathology which also includes diastolic dysfunction [32]; moreover, it has been proved that serum biomarkers of fibrosis including matrix metalloproteinases and tissue inhibitor of metalloproteinases are elevated in both cardiac and hepatic fibrosis [33]. Future studies should aim investigate the pathophysiological mechanisms behind the association of diastolic dysfunction and liver stiffness.

### Study Limitations

This study assessed a small number of patients without a placebo-controlled group after a short period of dapagliflozin therapy; however, the sample size estimation pointed to an appropriate sample size for the study’s primary end-point.

## 5. Conclusions

Although the mechanisms by which this molecule improves diastolic dysfunction are not fully understood, this research showed significant associations between the remission of DD1 and improvement of liver stiffness, increase of eGFR, increase of HbA1c, and EPI volume, regardless of age, BMI, or abdominal visceral adiposity. A difference of more than 0.4 kPa in the stiffness score predicts DD1 remission with a sensitivity of 71% and specificity of 70%.

A longer diabetes duration is associated with a decrease in the likelihood of DD1 duration. We can predict the remission of DD1 in T2DM patients with a diabetes duration of ≤ 8 years treated for one year with dapagliflozin with a sensitivity of 68.7% and specificity of 81.8%.

## Figures and Tables

**Table 1:** The dynamics of studied variables.

Variable	Baseline	At Six-Month Follow-Up	*p*
Glycemia (mg/dL) ^b^	213.1 ± 69.5	152.8 ± 35	<0.001 *
Total cholesterol (mg/dL) ^b^	197.1 ± 61.2	191.1 ± 46.5	0.07
Triglycerides (mg/dL) ^a^	170 (5; 887)	150 (58; 1397)	0.4
LDLc (mg/dL) ^b^	120.7 ± 47.1	107.4 ± 39.4	0.3
HDLc (mg/dL) ^a^	39 (17; 89)	42 (19; 90)	0.03 *
women ^b^	39.8 ± 11.8	45.1 ± 12.4	0.1
men ^a^	39 (17; 89)	41.5 (19; 90)	0.03 *
Uric acid (mg/dL) ^b^	4.9 ± 1.2	4.6 ± 1.1	0.1
eGFR (mL/min/1.73 m^2^) ^b^	86.1 ± 14.4	94.2 ± 13.2	0.007 *
HbA1c (%) ^b^	8.6 ± 1.1	7.9 ± 1.2	0.003 *
UACr (mg/g) ^a^	15.4 (5.5; 471.1)	16.7 (1.3; 928.3)	0.9
Weight (kg) ^b^	99.4 ± 4.7	94.7 ± 14.9	0.0001 *
women ^b^	94.5 ± 15.3	90.1 ± 15.7	<0.0001 *
men ^b^	103.6 ± 16.3	98.7 ± 13.3	<0.0001 *
BMI (kg/m^2^) ^b^	34.9 ± 4.7	33.3 ± 4.6	<0.0001 *
women ^b^	36.6 ± 5	35 ± 5.3	0.0002 *
men ^b^	33.4 ± 4	31.9 ± 3.5	<0.0001 *
waist (cm) ^b^	116 ± 11.8	114.3 ± 11.5	0.03 *
women ^b^	115.6 ± 10.1	115 ± 11	0.5
men ^b^	116.2 ± 13.2	113.7 ± 12.1	0.02 *
AST (u/L)	27 (12; 80)	21 (9; 45)	0.01 *
ALT (u/L)	47 (20; 174)	34.5 (21; 74)	0.002 *
Systolic BP (mmHg)	142.3 ± 21.5	137.8 ± 17	0.2
Diastolic BP (mmHg)	85 (65; 125)	85 (70; 105)	0.9
The epicardial fat thickness on echo cardiac (mm) ^b^	6 ± 1.3	4.2 ± 1.4	0.0001 *
women ^b^	6 ± 1.3	4 ± 1	<0.0001 *
men ^b^	6 ± 1.4	4.3 ± 1.7	0.0002 *
EPI volume (cm^3^) ^b^	37.6 ± 16.3	20.6 ± 7.3	<0.0001 *
women ^b^	35.8 ± 17	19.6 ± 5	0.0003 *
men ^b^	9.5 ± 16.1	21.4 ± 8.8	<0.0001 *
L4 visceral fat thickness (mm) ^b^	64 ± 15.8	65.4 ± 14.4	0.6
women ^b^	69.1 ± 14.5	68.7 ± 13.9	0.8
men ^b^	61.1 ± 17.1	62.8 ± 14.5	0.3
L4 fat volume (cm^3^) ^b^	37.8 ± 29.8	41.9 ± 18.5	0.4
women ^b^	35.5 ± 27.6	45.9 ± 17.4	0.07
men ^b^	42.9 ± 31.5	38.2 ± 19.1	0.5
Mediastinal fat thickness (mm) ^b^	24.4 ± 6.8	27.8 ± 6.9	0.02 *
women ^b^	25 ± 5.1	26.5 ± 6.5	0.1
men ^b^	25.8 ± 7.6	28.9 ± 7.1	0.1
CAP (dB/m) ^a^	368 (240; 400)	310 (190; 400)	0.0003 *
women ^b^	362.6 ± 31.7	316.1 ± 52	0.001 *
men ^a^	373 (240; 400)	310 (190; 400)	0.03 *
Fibroscan (liver stiffness) (kPa) ^a^	7.2 (3.5; 43.8)	6.7 (3.3; 15.3)	0.09
women ^a^	7.4 (3,5; 43.8)	7.3 (3.4; 15.3)	0.3
men ^a^	6.8 (3.9; 20)	6.2 (3.3; 12.1)	0.1

* The differences are statistically significant; ^a^ Continuous numerical variables with nonparametric distribution expressed as median and [interquartile range]; ^b^ The results of the variables with normal distribution are expressed as mean ± standard deviation; LDLc = low density lipid cholesterol; HDLc = high density lipid cholesterol; eGFR = estimated glomerular filtration rate; HbA1c = glycated hemoglobin; UACr = urinary albumin to creatinine ratio; BMI = body mass index; AST = aspartate aminotransferase; ALT = alanine aminotransferase; BP = blood pressure; EPI = epicardial adipose tissue; L4 = the forth vertebrae; CAP = controlled attenuation parameter; Fibroscan = liver stiffness score.

**Table 2:** Predictors associated with the remission of diastolic dysfunction (DD1).

Variable	Exp (β)	*p*
ΔHbA1c (%) **	1.85	0.03 *
ΔBMI (kg/m^2^) **	0.61	0.06
ΔGFR (mL/min/1.73 m^2^) **	0.91	0.02 *
Age (years) **	0.96	0.3
Diabetes duration (years) **	0.81	0.01 *
Δ liver stiffness (kPa) ***	1.24	0.04 *
ΔCAP (dB/m) ***	0.99	0.4

* The results are statistically significant; ** Univariate regression models; *** Logistic regression models.

**Table 3:** Risk analysis for DD1 remission depending on the predominant type of adipose tissue in study patients.

	Subcutaneous Adipose Tissue Predominance		Visceral Adipose Tissue Predominance	
	At Six Months	At One Year	At Six Months	At One Year
Relative risk	0.41	1.25	2.4	0.79
CI 95%	0.15–1.08	0.77–2.03	0.9–6.3	0.49–1.28
statistic z	1.791	0.932	1.7	0.93
*p*	0.07	0.35	0.07	0.3
Odds ratio	0.27	2.28	3.61	0.43
CI 95%	0.06–1.21	0.5–10.44	0.8–15.8	0.09–1.99
statistic z	1.698	0.932	1.6	1.066
*p*	0.08	0.28	0.08	0.28

CI = confidence interval.
